# Intra-articular injection of the anti-inflammatory compound LMWF-5A in adults with severe osteoarthritis: a double-blind prospective randomized controlled multi-center safety and efficacy trial

**DOI:** 10.1186/s13037-018-0158-0

**Published:** 2018-06-18

**Authors:** Kristin Salottolo, Brian Cole, David Bar-Or

**Affiliations:** 1 Trauma Research Department, Swedish Medical Center, 501 E. Hampden Ave Rm 4-454, Englewood, CO 80113 USA; 2 Department of Orthopedics, Rush University Medical Center, 1653 W. Congress Parkway, Chicago, IL 60612 USA; 3 Ampio Pharmaceuticals, Inc., 373 Inverness Parkway, Englewood, CO 80112 USA

**Keywords:** Safety, Efficacy, Osteoarthritis, Intra-articular injection

## Abstract

**Background:**

There are limited efficacious treatment options for severe osteoarthritis of the knee (OAK). The Low Molecular Weight Fraction of 5% human serum Albumin (LMWF-5A) is in development to treat severe OAK. This study evaluated the safety and efficacy of LMWF-5A for the signs and symptoms of OAK.

**Methods:**

This 12-week randomized, double-blind, controlled clinical trial was conducted at thirteen sites across the United States. Patients with symptomatic, severe OAK (Kellgren-Lawrence grade 4 disease) who were fully ambulatory and had no other conditions interfering with the study knee were randomized to a single 4 ml intra-articular injection of LMWF-5A or saline, randomized 6:1. The primary endpoint was Outcome Measures in Rheumatology-Osteoarthritis Research Society International (OMERACT-OARSI) responder rate (%), examined with a one-sided exact binomial test compared to a clinically meaningful response rate of 30%. Efficacy of LMWF-5A was also evaluated as controlled responder (%), defined as 20% improvements in both pain and function, compared to historical saline control from three previous trials. Safety was examined as the incidence and severity of adverse events (AEs). This trial was registered (clinicaltrials.gov identifier: NCT03182686).

**Results:**

In total, 168 patients were randomized; 144 subjects treated with LMWF-5A were analysed. Overall, 71% (95% CI: 63.4%–78.3%) of subjects treated with LMWF-5A met the OMERACT-OARSI responder criteria, exceeding the 30% threshold (*p* < 0.001). There were also significantly more responders at week 12 in the LMWF-5A arm than historical saline control (65% vs. 43%, *p* < 0.001). There were no drug-related serious AEs reported and no deaths or withdrawals due to adverse events.

**Conclusion:**

LMWF-5A provides relief for the signs and symptoms of severe osteoarthritis, and may be an alternative therapeutic treatment option for patients with severe osteoarthritis of the knee.

## Background

Osteoarthritis (OA) is an incurable and progressive disorder of the joints involving degradation of the intra-articular cartilage, joint lining, ligaments, and bone. OA affects up to 27 million adults in the US alone [1]. The incidence of developing osteoarthritis of the knee (OAK) over a lifetime is approximately 45%, and this number is expected to grow because OAK is associated with age, obesity, and diabetes [1]. The primary clinical symptoms are pain and functional impairment leading to loss of mobility [2]. The severity of OAK is typically defined in stages as noted by Kellgren-Lawrence (KL) grading: Grade 0 (normal knee; no osteophytes or joint space narrowing), Grade 1 (possible osteophytic lipping and doubtful narrowing of joint space), Grade 2 (definite osteophytes and possible narrowing of joint space), Grade 3 (moderate multiple osteophytes, definite narrowing of joint space and some sclerosis, and possible deformity of the bone ends) and Grade 4 (large osteophytes, marked narrowing of joint space, severe sclerosis, and definite deformity of bone ends) [3].

Practice guidelines recommend the use of nonsteroidal anti-inflammatory drugs (NSAIDs), cyclooxygenase-2 inhibitors, or acetaminophen for the pharmacologic management of pain in OAK (all KL grades) [4]. Steroids and hyaluronic acid injections have been approved for the treatment of OAK. However, they are not without controversy due to varying evidence of efficacy [4, 5]. Further, these products received approval for clinical studies where patients with KL grade 4 disease were excluded, and there are no published studies examining their use exclusively in the KL grade 4 population. As such, there is an unmet therapeutic need for patients with severe OAK who live with debilitating pain as well as functional and activity limitations. Patients with severe OAK routinely require a total knee replacement due to a lack of effective treatment options. Over 700,000 total knee replacements are performed each year in the United States [6]. This number is expected to spiral to over 3.5 million procedures by 2030, in part because the aging patient population and increasing obesity rates have resulted in increasing OA prevalence and severity [6].

LMWF-5A, the < 5 kilodalton (kDa) ultrafiltrate of 5% human serum albumin, is a novel non-steroidal, anti-inflammatory biologic agent which has been shown to effectively reduce pain in patients with severe OAK when administered as a single intra-articular (IA) injection [7, 8]. The molecular components and mechanisms of action of LMWF-5A have previously been described [9–13].

LMWF-5A is in development to provide relief for the signs and symptoms of severe OAK. Prior trials evaluating LMWF-5A were conducted in a population with all stages of symptomatic OAK (KL grades 2–4), and examined pain as the primary endpoint. The objective of this study was to confirm the efficacy of LMWF-5A in a population of patients with severe OAK (KL grade 4), evaluating both the signs and symptoms of OAK.

## Methods

### Design and setting

This was a randomized controlled trial to evaluate the efficacy of a single injection of 4 ml LMWF-5A in patients with severe OAK (KL grade 4). Patients were randomized 6:1 to LMWF-5A or saline. The trial was performed in accordance with the principles of Good Clinical Practice guidelines, in compliance with the CONSORT standards, received institutional review board (IRB) approval across all thirteen sites, and was registered prior to patient recruitment (Clinicaltrials.gov identifier: NCT03182686). Patients were enrolled at thirteen sites within the United States between June 19, 2017 and September 15, 2017 with follow-up and study completion through December 7, 2017. Updates to the study protocol that were made after the study commenced but prior to study completion, include: changes to secondary endpoints to include the endpoints listed below; Inclusion criteria of at least moderate functional impairment (score > 1.5 Western Ontario and McMaster Universities Arthritis (WOMAC®) function subscale) to ensure patients presented with signs and symptoms of OAK.

### Test product

The test product is LMWF-5A, which is the < 5 kilodalton (kDa) ultrafiltrate of 5% human serum albumin. The starting material, commercial HSA, was subjected to centrifugation/ultrafiltration under sterile conditions. The ultrafiltrate, containing species with a molecular weight less than 5000 Da, was separated. The ultrafiltrate contains aspartyl-alanyl diketopiperazine (DA-DKP, approximately 50–200 μM) and the excipients (i.e. sodium caprylate and sodium acetyltryptophanate). The ultrafiltrate was transferred for aseptic filling to afford sterile drug product. DA-DKP has been shown to have multiple anti-inflammatory and immune modulating effects [9–13], and is believed to be one of the active ingredients in the pharmacological effects of commercial HSA.

### Participants

Eligible subjects had x-ray findings demonstrating KL grade 4 OAK, with at least moderate pain and functional impairment (defined as a score of at least 1.5 on the 5-point Likert WOMAC osteoarthritis Index 3.1 pain and function subscales), with the ability to discontinue NSAID use at screening for the duration of the study, be fully ambulatory, have no known clinically significant liver abnormality, and between 40 to 85 years old. Subjects also had to have minimal or no pain in the contralateral knee (pain < 1.5 on the 5-point Likert WOMAC pain subscale).

Exclusion criteria included: a history of allergic reactions to albumin and its excipients; any human albumin treatment 3 months before randomization; concurrent arthritic conditions or other conditions interfering with the free use and evaluation of the index knee (e.g. chondromalacia, presence of tense effusions, acute fractures, history of aseptic necrosis or joint replacement in the affected knee, inflammatory or crystal arthropathies); severe hip OA ipsilateral to the index knee; major surgery within 12 months prior to screening; pregnancy; any treatment targeting OAK started or changed 4 weeks before randomization; use of other IA-injected medications at least 12 weeks (hyaluronic acid) or 4 weeks (steroids) prior to baseline; use of opioids, NSAIDs, topical treatments, significant anticoagulant therapy, immunosuppressants, corticosteroids, or systemic treatments that may interfere with study assessments.

### Randomization and blinding

Randomization was developed and maintained by an independent statistician. Patients were randomized 6:1 to receive a single 4 mL intra-articular injection of LMWF-5A or saline (0.9% sodium chloride) into the knee joint space (inferior lateral to the patella). Study drugs were provided in vials labeled with double-panel labels blinded for drug content. Lidocaine injection was not allowed preceding the administration of study drug. Acetaminophen was allowed as pain analgesia during the study as 500 mg tablets every 4 h, as required. The Sponsor, the investigators, and all study staff having a role in the day-to-day conduct of the study remained blinded to treatment.

### Assessments and endpoints

The clinical effects of LMWF-5A was evaluated during in-clinic visits at baseline, 6 and 12 weeks, and telephone contacts at 2 and 10 weeks, using the WOMAC osteoarthritis Index 3.1 5-point Likert score and the Patient’s Global Assessment of disease severity (PGA). Clinical benefit was evaluated using the Outcome Measures in Rheumatology (OMERACT) - Osteoarthritis Research Society International (OARSI) (OMERACT-OARSI) responder criteria. The OMERACT-OARSI response uses the WOMAC and PGA scales as assessed during the in-office visits and phone contacts.

Safety was assessed by recording adverse events and concomitant mediations (all follow-up contacts), physical examination and vital signs (Baseline, Weeks 6 and 12), and laboratory tests (Screening and Week 12).

The primary endpoint was OMERACT-OARSI response, defined using scenario D as follows: a) Large improvement in WOMAC A pain or in WOMAC C function ≥50% and absolute change of ≥1 points, or b) moderate improvements in at least 2 of 3 response domains: i) WOMAC A pain ≥20% and absolute change ≥0.5; ii) WOMAC C function ≥20% and absolute change ≥0.5; iii) PGA ≥ 20% and absolute change ≥0.5.

Secondary efficacy endpoints included: a) controlled responder, defined as an improvement in both WOMAC A pain and WOMAC C function of ≥20% and an absolute change of 0.5 points; b) PGA responder, defined as a ≥ 20% improvement and absolute change ≥0.5 points in PGA.

### Sample size and comparison groups

Two comparators were used for evaluation of efficacy of LMWF-5A. First, a 30% response to treatment was considered demonstration of a meaningful response to treatment of at least minimum efficacy. While the amount of improvement required may not be definitively established and is stated to be between 20%–40% based on best available evidence [14], the 30% threshold was selected based on Initiative on Methods, Measurement, and Pain Assessment in Clinical Trials (IMMPACT) recommendations demonstrating 30% represents moderate improvements in chronic pain intensity in pain trials [15]. A 20–30% result is expected in OA when evaluated by the OMERACT-OARSI scenario D criteria [16]. The American College of Rheumatology (ACR) also uses a 30% response to treatment in the established ACR30 criteria, which evaluates six core domains including PGA and physical function [17].

The hypotheses were tested as follows: H0: π ≤ π0 versus HA:π > π0, where the null hypothesis was a 30% response to treatment (π0). A sample size of 146 patients in the LMWF-5A arm was calculated to have power greater than 90% when the anticipated proportion of responders under the alternative hypothesis was 45%.

Second, efficacy of LMWF-5A was evaluated versus a historical saline-control, defined a priori as all patients with KL grade 4 disease who were randomized to saline arm (*n* = 223) from three previous studies comparing LMWF-5A and saline when administered as a single injection [8]. The current trial and all previous trials [8] were identical for selection criteria, treatment arms, blinding, randomization (1:1), and safety and efficacy assessments. While the study was powered to compare LMWF-5A to a clinically relevant threshold response, the comparison to historical saline was used to ensure a meaningful and quantitative assessment of treatment effect. The Food and Drug Administration allows comparison to historical control in its clinical trial guidance document [18]. There are currently no approved active controls (no IA-injected therapies approved in the severe KL grade 4 OAK population), and no available placebo controls. While saline has historically been used as a control in IA trials, recently saline has demonstrated a pronounced therapeutic effect when administered as an IA injection into the knee [19–21].

### Statistical analysis

Statistical analyses were performed using SAS® software, version 9.3 (SAS Institute, Cary, NC). Efficacy endpoints were analyzed in the intent-to-treat population (as randomized). For the primary effectiveness endpoints (WOMAC A, WOMAC C, and PGA), missing Week 12 values were imputed using the Worst Observation Carried Forward (WOCF) method, which is a more conservative approach to imputation than the last observation carried forward (LOCF) approach when patients are expected to improve over time, and is frequently used in analgesia drug trials. All endpoints were defined a priori.

The efficacy endpoints were tested in a hierarchical manner, as the primary (OMERACT-OARSI responder), and then secondary endpoints of controlled responder, then PGA responder, then controlled responder compared to historical saline. The primary and secondary efficacy endpoints were reported as proportions (%). A one-sided exact binomial test was used for comparison of LMWF-5A to the 30% threshold (OMERACT responder, controlled responder, PGA responder); statistical significance was set at *p* value < 0.025. The Fisher’s exact test was used for comparison of LMWF-5A to historical saline for controlled responders; statistical significance was set at *p* value < 0.05.

Adverse events (AEs) were examined in all patients who were randomized. Missing or incomplete AE data was assumed to be a severe, related AE. Adverse events were tabulated for incidence and severity; severity was defined as mild (symptom barely noticeable to the subject), moderate (symptom is of sufficient severity to make the subject uncomfortable), and severe (symptom causes severe discomfort, daily activities are significantly impaired or prevented). Serious adverse events (SAEs) were defined as untoward medical occurrences resulting in death, in-patient hospitalization, persistent or significant disability/incapacity, or were life-threatening.

## Results

**Fig. 1:**
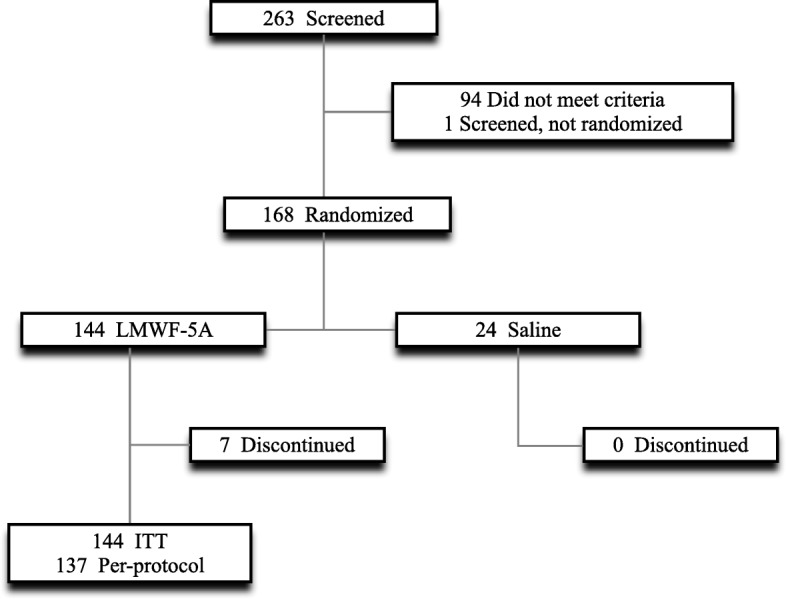
CONSORT flowchart

Of 168 subjects, 144 subjects were randomized to LMWF-5A and 24 subjects were randomized to saline (Fig. 1). Patients were randomized across 13 sites, with no site effect observed (*p* = 0.82). All 144 subjects treated with LMWF-5A were included in the analysis. Missing data accounted for 2.0% of all efficacy assessments, and 4.2% (*n* = 7) of patients had WOCF values imputed for the primary endpoint at week 12.

Baseline data is shown in Table 1. Just over half of subjects were female (52%), the average age was 63 years, and the average baseline pain was 2.5 (0.6) on a 0–4 scale. There were no clinically meaningful imbalances between LMWF-5A, saline, and historical saline in demographics or baseline WOMAC subscores.

**Table 1:** Demographics and baseline characteristics

Mean (SD)	LMWF-5A (*n* = 144)	Saline (*n* = 24)	Historical saline (*n* = 223)
Female sex, % (n)	52.8 (76)	50.0 (12)	51.6 (113)
Age, years	62.7 (9.6)	64.0 (6.3)	63.1 (8.6)
BMI (lb/in2)	32.7 (6.4)	31.4 (6.3)	34.4 (7.9)
Caucasian race	77.1 (111)	66.7 (16)	88.8 (198)
Patient global assessment (PGA)	2.8 (0.8)	2.7 (0.8)	2.6 (0.8)
Western Ontario and McMaster Universities Osteoarthritis Index (WOMAC)			
WOMAC Pain	2.5 (0.6)	2.4 (0.4)	2.3 (0.6)
WOMAC Stiffness	2.8 (0.7)	2.8 (0.6)	2.5 (0.7)
WOMAC Function	2.6 (0.5)	2.5 (0.5)	2.4 (0.6)

### Primary efficacy endpoint

Overall, 71% (95% CI: 63%–78%) of subjects treated with LMWF-5A met the OMERACT-OARSI responder criteria, which exceeded the 30% threshold for clinical benefit (*p* < 0.001), Table 2. Responders experienced, on average a 53% decrease in pain as measured by WOMAC A and a 50% improvement in function as measured by WOMAC C and a 45% improvement in quality of life as measured by PGA. Treatment with LMWF-5A resulted in a significant response using OMERACT-OARSI criteria at all assessed time points (*p* < 0.001 for all, Table 2).

**Table 2:** Summary of efficacy

Responder rate, % (95% CI)	OMERACT-OARSIResponder	Controlled Responder	PGA responder
Week 2	66.0 (58.2–73.7)	54.2 (46.0–62.3)	66.0 (58.2–73.7)
Week 6	66.0 (58.2–73.7)	58.3 (50.3–66.4)	61.8 (53.9–69.7)
Week 10	72.2 (64.9–79.5)	61.8 (53.9–69.7)	72.9 (65.7–80.2)
Week 12	70.8 (63.4–78.3)	64.6 (56.8–72.4)	66.0 (58.2–73.7)

*CI* confidence interval, *OMERACT-OARSI* Outcome Measures in Rheumatology (OMERACT) - Osteoarthritis Research Society International (OARSI)*, PGA* Patient Global

Assessment. Efficacy endpoints were analyzed with one-sided exact binomial test compared to null hypothesis of 30% responder rate. *P* < 0.001 for all responder rates at all time points

### Secondary efficacy endpoints

The percent of subjects in the LMWF-5A arm that met the controlled responder criteria was 65% (95% CI: 57%–72%), which was significantly greater than the threshold of 30% (*p* < 0.001), Table 2. Further, the proportion of subjects that met controlled responder at weeks 2, 6, 10, 12 was significantly greater than the established threshold, at all time points (*p* < 0.001, Table 2).

The percent of subjects treated with LMWF-5A that were PGA responders was 66% (58%–74%), exceeding the 30% threshold (*p* < 0.001), Table 2. The proportion of subjects that met PGA responder at weeks 2, 6, 10, 12 was significantly greater than the established threshold, at all time points (*p* < 0.001 for all, Table 2).

When compared to 223 historical saline controls, the percent of subjects that met the controlled responder criteria at week 12 was significantly greater in the LMWF-5A arm than the historical control arm (65% vs. 43%, *p* < 0.001). The percent of subjects meeting controlled responder criteria was also significantly greater for LMWF-5A vs. historical control at week 10 (62% vs. 47%, *p* = 0.007) and week 6 (58% vs. 43%, *p* = 0.005), but was similar at week 2 (54% vs. 49%, *p* = 0.33).

### Safety

Adverse events were reported for 49 (34.0%) LMWF-5A-treated patients (Table 3). The most commonly occurring AE was arthralgia, reported in 19 (13.2%) patients treated with LMWF-5A. The majority of AEs were of minor or moderate severity and unrelated to treatment. There were no SAEs. AEs reported in this trial are in line with what has been reported in previous trials of LMWF-5A of over 2000 patients (Table 3). In both trials the most commonly occurring AE was arthralgia, which is considered a typical AE for IA injection trials in patients with OAK.

**Table 3:** Summary of Adverse Events (AEs)

Endpoint	LMWF-5A KL grade 4 (*n* = 144)	LMWF-5A KL grade 2–4 (*n* = 1076)	Saline KL grade 2–4 (*n* = 931)
One or more AE	49 (34.0%)	381 (35.4%)	386 (41.5%)
One or more related AE	10 (6.9%)	11 (1.0%)	19 (2.0%)
AE by severity^a^			
mild	25 (17.4%)	264 (24.5%)	253 (27.2%)
moderate	22 (15.3%)	162 (15.1%)	190 (20.4%)
severe	2 (1.4%)	35 (3.3%)	32 (3.4%)
Serious AE (SAE)	0 (0%)	15 (1.4%)	20 (2.1%)
AE leading to withdrawal	0 (0%)	0 (0%)	0 (0%)
AE resulting in death	0 (0%)	0 (0%)	0 (0%)

^a^Subjects could have AEs in more than one severity category. KL, Kellgren Lawrence

## Discussion

This trial was designed to follow on from the completed single-injection LMWF-5A studies previously described [8] and confirmed the efficacy of an IA injection of LMWF-5A. This trial explored the clinical impact of LMWF-5A on the signs and symptoms of severe OAK using the criteria developed by OMERACT-OARSI. Using these established criteria, 71% of patients treated with LMWF-5A responded to treatment, with clinically meaningful improvements in pain and function, supported by improvements in overall global assessment of disease. There were no drug-related serious AEs associated with treatment with LMWF-5A. The safety and tolerability profile of LMWF-5A is consistent with previous studies. To date, treatment with LMWF-5A has resulted in no reported drug-related SAEs.

Clinical trials that evaluate symptom-modifying effects of treatment often examine composite endpoints that incorporate the main symptoms of OAK (pain and functional limitations). One of the most widely established composite endpoints is the OMERACT-OARSI responder [16] because it addresses clinically important and statistically significant improvements. In prior trials that reported OMERACT-OARSI responders, rates were reported to be 50–70% with active treatment across all severity of OAK (KL grades 1–4) [22–27]. The favorable responder rate of 71% observed in this study was in a subset with KL grade 4 OAK. Patients with severe disease have been shown to have a lower response to treatment than patients with less severe OAK [28].

There are limitations. First, patients were randomized to saline to maintain blinding, but since it is not a true placebo, [19–21] it was not used as the comparator or powered to make inference about patients receiving saline. This trial was designed using FDA guidance to address the methodological issues in trial design where there is the absence of a true available control, such as when there are no licensed or approved active comparators in this distinct OAK population and where the previously used placebo control has been shown to be therapeutic. Second, imaging or biomarker data were not collected, and all outcomes were based on patient-reported outcome measures. Third, study participants were followed until week 12. An open label extension study is currently being conducted to determine the clinical benefit of LMWF-5A through 52 weeks (Clinicaltrials.gov identifier: NCT03349645).

## Conclusions

LMWF-5A appears to be an alternative therapeutic treatment option for patients with severe OAK, by safely and effectively reducing the signs and symptoms of this debilitating symptomatic disease.

## Abbreviations

ACR: American College of Rheumatology
AE: Adverse events
IA: Intra-articular
IMMPACT: Initiative on Methods, Measurement, and Pain Assessment in Clinical Trials
KL: Kellgren Lawrence
LMWF-5A: Low Molecular Weight Fraction of 5% human serum Albumin
LOCF: Last observation carried forward
NSAIDS: Nonsteroidal anti-inflammatory drugs
OA: Osteoarthritis
OAK: Osteoarthritis of the knee
OARSI: Osteoarthritis Research Society International
OMERACT: Outcome Measures in Rheumatology
PGA: Patient global assessment
WOCF: Worst Observation Carried Forward
WOMAC: Western Ontario and McMaster Universities Arthritis Index
